# 

**Published:** 1859-07

**Authors:** 


☞ Dr. A. G. Thomas has resigned the Chair of Physiology in the Oglethorpe Medical College, and Dr. Wm. Hauser, of Jefferson County, Ga., has been appointed to fill the vacancy

